# Axonopathy: mechanisms and potential therapeutic targets for neurodegenerative diseases

**DOI:** 10.1186/s40035-026-00543-7

**Published:** 2026-03-24

**Authors:** Ruinan Shen, Kijung Sung, Jianqing Ding, Chengbiao Wu

**Affiliations:** 1 Institute of Neurology, Ruijing Hospital, Shanghai Jiao Tong University School of Medicine, Shanghai, China; 2 Department of Neurosciences, University of California San Diego, La Jolla, San Diego, CA USA; 3 Institute of Aging & Tissue Regeneration, Renji Hospital, School of Medicine, Shanghai Jiao Tong University, Shanghai, China; 4 Department of Neurosciences, University of California San Diego, 9500 Gilman Dr, San Diego, CA 92093 USA

**Keywords:** Axon, Axonal transport, Neurodegeneration, Neurotrophic factors, Alzheimer’s disease, Parkinson’s disease, Huntington’s disease, Amyotrophic lateral sclerosis, Mitochondria, Autophagy

## Abstract

Axons are unique structural and functional features of nerve cells, which play a critical role in regulating neuronal homeostasis. Dysfunction and degeneration of axons (axonopathy) has been established as an early and prominent contributing mechanism to the pathogenesis of neurodegenerative diseases including Alzheimer’s disease, Parkinson’s disease, Huntington’s disease, and amyotrophic lateral sclerosis. In this review, we briefly summarize the structure and function of axons, and highlight recent advances in the understanding of the role of axons in health and disease. We argue that axons are a potential target for developing novel therapies for neurodegenerative diseases.

## Introduction

The intricate biology and the breadth of pathophysiology of the axon positions it as a critical nexus of vulnerability in neurodegenerative diseases. Disruptions in axonal structure and function contribute to early cellular pathologies in neurodegeneration. The endolysosomal system serves as a fundamental hub where numerous genetic risk factors for diseases like Alzheimer’s (AD) and Parkinson's (PD) converge, initiating a cascade of downstream pathologies including impaired neurotrophic signaling and protein misprocessing. These intra-axonal events can also be impacted by non-neuronal residents in the brain. Astrocytes, microglia, and oligodendrocytes are not merely passive bystanders but active participants in maintaining axonal health. When dysregulated, they drive the demise of the axon. By connecting the internal axonal crisis such as transport impairment with the external impact of glial cells, this review aims to synthesize a unified perspective on a core pathogenic circuit in neurodegeneration—a focus that distinguishes it from broader reviews on axonal transport and highlights mechanistic bridges between neuronal and non-neuronal contributions to disease.

## Axons: from structure to function

### Axonal structure

#### Length

The axon is a unique, often extremely long neuronal projection that can extend over a meter in humans, creating a challenge for neuronal maintenance and communication. Its specialized structure is fundamental to its function.

#### Axon initial segment (AIS)

AIS, an unmyelinated subdomain proximal to the soma, serves as a critical interface between the somatodendritic compartment and the axon [1]. It exhibits remarkable activity-dependent structural plasticity, with remodeling of its length and ion channel distribution in response to neuronal activity to maintain the homeostatic balance—evidenced by adaptations to chronic depolarization [2]. This plasticity is finely regulated by GABAergic inputs, particularly from chandelier cells (ChCs), which form specialized synapses on the AIS of projection neurons to suppress action potential initiation [3, 4]. Calcium channel-dependent mechanisms further modulate AIS excitability in response to altered GABAergic signaling [5]. Structurally, the AIS functions as a selective diffusion barrier, comprising microtubule fascicles and periodic actin rings that restrict the lateral movement of membrane proteins such as SK channels and NKCC1a/1b, thereby preserving axonal identity and functional integrity [6, 7]. These microtubule bundles not only physically confine the retrograde diffusion of pathogenic proteins like tau—a function modulated by divalent cations (e.g., Mg^2^^+^, Ca^2^^+^) and implicated in neurodegeneration when impaired [6]—but also provide a directional track for dynein-mediated retrograde transport through their uniformly oriented "plus-end-out" arrangement. This polarized microtubule array, maintained by γ-TuRCs and CLASP proteins, ensures trafficking specificity by selectively excluding dendritic cargos [8]. Functionally, the AIS actively collaborates with the retrograde transport machinery via adaptors such as DOK6, which specifies the retrograde signaling of particular neurotrophin receptors (e.g., TrkC, Ret), and SNAPIN, whose phosphorylation at Thr-14 fine-tunes mitochondrial retrograde transport [9, 10]. Although composed of highly dynamic individual microtubules, the AIS array exhibits collective "dynamic stability", enabling local renewal while upholding global barrier function—a property reinforced by scaffolds like Ankyrin G (AnkG) to balance transport efficiency with selective filtering [11–13]. Together, these mechanisms establish the AIS not as a passive barrier, but as an intelligent "gatekeeper" that integrates molecular interactions, motor protein regulation, and dynamic structural remodeling to ensure precise neuronal compartmentalization.

#### Cytoskeleton

Axonal cytoskeleton acts as a directional conduit for organelle transport to support the growth of axons and dendrites. The dynamics of axonal cytoskeleton is regulated by multiple mechanisms, including mechanical stimulation, phosphorylation, and gene expression. External mechanical forces, as demonstrated by magnetic nano-pulling experiments, can increase the axonal microtubule density, thereby promoting the accumulation of organelle vesicles and local translation processes, ultimately enhancing the signal-processing capacity of axons [14]. Phosphorylation and post-translational modifications contribute significantly to cytoskeletal regulation. The phosphorylation profile dynamics differs markedly between axons and cell bodies in response to physiological neuronal stimulation, indicating axon-specific regulatory mechanisms. For example, Ca^2^^+^ surges can cause periodic disruption of the cytoskeleton [15]. Additionally, aberrant oxidation of protein 4.1B caused by mitochondrial dysfunction, disrupts the anchoring of neurofilaments to paranodal structures, such as the Caspr/contactin/NF155 complex, thereby compromising the axonal integrity [16].

#### Myelin sheath and nodes of Ranvier

Many axons are ensheathed by myelin, a lipid-rich extension of glial cells. The myelin sheath is periodically interrupted by nodes of Ranvier. These nodes concentrate ion channels to enable saltatory conduction, drastically increasing the speed of action potential propagation [17] and serving as sites for neuron-glia communication [18]. Recent research has revealed myelin as a dynamic structure with activity-dependent plasticity. Processes such as localized calcium signaling and metabotropic glutamate receptor 5 activation promote myelin elongation, a mechanism critical for learning and memory consolidation [19]. Concurrently, the precise formation of nodes of Ranvier depends on coordinated axon-intrinsic and glial-extrinsic mechanisms, through which glial cells recognize axonal surface molecules like neurofascin to regulate myelin termination and establish nodal domains [20]. These interrelated mechanisms underscore the active role of myelination in neural circuit adaptation and function.

The unique structural characteristics of axons, from the extraordinary diversity in length to elegant myelin sheath/nods of Ranvier, and to sophisticated AIS, point to the critical roles of axons in the development and maintenance of nervous systems.

### Axonal function

#### Bidirectional axonal transport

Bidirectional axonal transport is essential for neuronal homeostasis, enabling the exchange of organelles, proteins, lipids, and RNAs between the soma and distant synaptic terminals [21]. Anterograde transport, mediated by kinesin superfamily motors, delivers newly synthesized materials to support synaptic structure and function [22], while retrograde transport, driven by cytoplasmic dynein, returns signaling factors and damaged organelles to the soma for nuclear signaling and degradation [23]. The dynein complex requires precisely coordinated interactions with its cofactor dynactin and regulatory proteins such as LIS1 and NDEL1; however, the mechanisms governing this coordination during long-distance trafficking remain incompletely understood [24]. Local protein synthesis in distal axons contributes to this signaling network, exemplified by the local translation of the proNGF receptor p75, whose subsequent retrograde transport can trigger somatic degeneration programs [25]. Metabolic regulation further fine-tunes the transport efficacy: the maintenance of NAD⁺ redox potential in distal axons—dependent on NMNAT2 activity—is critical for vesicular glycolysis and fast transport, whereas sterile alpha toll/interleukin receptor motif containing-1 (SARM1)-dependent NAD⁺ depletion disrupts trafficking and promotes axon degeneration [26]. Examples of molecules involved in this sophisticated transport system are key trafficking adaptors, including DOK6, which specifically facilitates retrograde transport of TrkC/Ret neurotrophic signaling complexes [9], and SNAPIN, whose phosphorylation at Thr-14 regulates mitochondrial retrograde movement [10].

Advanced imaging approaches have provided unprecedented insights into these transport dynamics. Two-photon microscopy in live axons has revealed that autophagic vesicles undergo predominantly retrograde transport (85%), moving at higher speeds than their anterograde counterparts [27]. Concurrently, nonlinear imaging of nanocrystals has resolved fundamental "run-and-pause" motility patterns at high spatiotemporal resolution, enabling the detection of subtle transport abnormalities in preclinical disease models [28, 29]. The efficiency of mitochondrial transport, a process visualized by these techniques, has been established as a critical determinant of regenerative capacity following neural injury [30]. Collectively, these technological advances not only delineate the physiological dynamics of axonal trafficking but also provide sensitive tools for identifying early pathological disruptions in neurodegenerative conditions.

#### Axonal trafficking and endocytic machinery

The endolysosomal pathway, which progresses from Rab5-positive early endosomes to Rab7-marked late endosomes/lysosomes, is essential for cargo degradation and calcium regulation in axons [31, 32]. Recent studies have revealed that these vesicles also carry RNA granules, enabling localized protein synthesis within axons [33]. During retrograde transport, endosomes undergo acidification and maturation, with fully active lysosomes primarily localized to the soma. Critically, this trafficking system mediates neurotrophic and injury-related signaling: internalized neurotrophic factor receptors (e.g., Trk, p75) are sorted through Rab5/Rab7-endosomes, activating downstream pathways such as PI3K (phosphoinositide 3-kinase)-Akt, MAPK, and PLCγ (phospholipase C-gamma) to regulate neuronal survival, growth, and synaptic maintenance [34–36]. Additionally, Rab5-positive early endosomes modulate JNK (c-Jun N-terminal kinase)-mediated death signaling [37]. Disruption of this finely regulated endosomal transport represents an early pathological event in neurodegenerative diseases [38].

Recent studies on endocytic machinery have revealed unique characteristics of the AIS. In the axonal shaft, clathrin-mediated endocytosis operates differently from that in dendrites or presynaptic terminals. At the AIS, clathrin-coated structures interact with the sub-membranous periodic actin-spectrin scaffold, forming specialized endocytic sites; however, the precise mechanisms governing this process remain incompletely understood [39]. Furthermore, Schwann cells actively induce axonal clathrin-mediated endocytosis (CME) prior to myelination, locally activating the endocytic removal of various axonal cell adhesion molecules (CAMs). Failure to internalize these CAMs impedes myelination, underscoring the critical role of endocytic machinery in axon-glia interactions [40]. Technologically, endosome immunoprecipitation (Endo-IP) coupled with single-cell RNA sequencing of EEA1-positive endosomes has enabled the profiling of neuronal endocytic cargo (e.g., transmembrane proteins) and lysosomal protein composition. This approach can simulate neuronal depolarization states, providing a powerful tool for studying endocytic dynamics [41].

#### Axon and autophagy

Autophagosomes are primarily generated at axon terminals and undergo retrograde transport along microtubules toward the cell body for lysosomal degradation [42]. Notably, certain presynaptic endocytic proteins involved in synaptic vesicle recycling also participate in autophagic regulation, indicating a functional crosstalk between endocytic and autophagic pathways in axons [6, 27, 43]. During this transit, autophagosomes mature through repetitive fusion events, with transport efficiency declining significantly in aged neurons, leading to reduced acidified vesicles in distal axons and impaired autophagic flux [44]. Functionally, axonal autophagy clears damaged proteins, organelles (e.g., via mitophagy), and inflammatory factors to maintain axonal homeostasis, while also recycling nutrients under metabolic stress [45]. This degradative pathway is tightly regulated by transcription factors such as Lhx2—which suppresses Sema3C to promote axon regeneration [46]. Additionally, efficient autophagosome-lysosome fusion requires lysosomal acidification and regulators like PLEKHM1, with defects exacerbating α-synuclein (α-syn) pathology [47].

#### Axon and neuronal energetics

Axons have a high energy demand to sustain their functions. About 55% of total neuronal ATP is consumed at presynaptic terminals to maintain ionic gradients and support synaptic transmission [48]. To optimize energy allocation, axonal metabolism is compartmentalized: the AISs are activity-regulated, the myelinated segments rely on glial metabolic support, and the presynaptic boutons are specialized for synaptic signaling, all under the central regulation of NAD^+^ homeostasis [49]. Beyond action potentials, substantial energy is dedicated to molecular turnover, with local glycolysis coupled to structural dynamics like axonal growth and retraction [50, 51]. This intricate system is vulnerable, as aging disrupts the metabolic balance, contributing to connectome disintegration [52], while oligodendrocytes form "axon-oligodendrocyte metabolic units" via activity-dependent K^+^ signaling to provide critical lactate and glucose support [53].

Beyond its canonical role as an energy currency, ATP serves a crucial function as a biological hydro trope that maintains axoplasmic fluidity and prevents pathological protein aggregation. A drop in intracellular ATP concentration, such as that caused by mitochondrial inhibition, significantly increases axoplasmic viscosity and promotes liquid-phase separation and condensation of proteins linked to neurodegenerative diseases [54]. This novel role establishes ATP as a key regulator of the physicochemical state of the axonal cytoplasm, with its depletion directly contributing to the protein aggregation pathologies characteristic of neurodegeneration.

Mitochondria are pivotal in meeting these fluctuating demands. Their strategic distribution is ensured through transport, anchoring at high-demand sites by proteins like syntaphilin [55], and even local biogenesis in distal axons [56]. Once positioned, their function is maintained through dynamic fission—mediated by DRP1 (dynamin-related protein 1) with adaptors like MFF (mitochondrial fission factor) and MiD49/51 (mitochondrial dynamics proteins of 49 and 51 kDa) [57, 58]—and fusion, governed by mitofusins to mix contents and optimize ATP production [59]. This local ATP production is paramount not only for fueling biochemical reactions but also for preserving the soluble state of the presynaptic cytosol and its components, including synaptic vesicles and active zone proteins. Consequently, mitochondrial dysfunction leads to a rapid decrease in local ATP, triggering a phase transition in the cytosol that underlies the pathological aggregation observed in neurodegeneration [54]. To preserve quality, damaged components are removed via mitochondrial-derived vesicles [60] or through PINK1 (PTEN-induced kinase 1)-Parkin-mediated mitophagy for severe damage [61]. Notably, the aggregation propensity of Parkin itself is regulated by ATP-dependent liquid-phase separation, directly linking mitochondrial quality control mechanisms to the solubility of their own components. Dysregulation in any aspect of this mitochondrial life cycle from biogenesis and transport to dynamics and quality control—is fundamentally linked to neurodegenerative pathogenesis [62–65].

#### Axons and synapses

Axons form structurally and functionally diverse synapses that are precisely organized to support complex neural computation. Cerebellar granule cells, for example, extend ascending axons and parallel fibers that establish distinct excitatory synapse populations on Purkinje cells, with a single Purkinje cell integrating up to ~ 175,000 such inputs through subtype-specific plasticity mechanisms [66]. This synaptic heterogeneity extends to the spatial distribution of *en passant* synapses along axonal shafts, which require efficient delivery of presynaptic components, such as Bruchpilot in *Drosophila* olfactory axons, to maintain network connectivity and regulate behavior [67, 68]. At the ultrastructural level, synaptic nanotopography undergoes activity-dependent refinement, where mature synapses develop nanostructures that support rapid vesicle release, whereas sensory deprivation disrupts this developmental program [69]. Energetically, synapses represent axonal hot spots that depend on strategically anchored mitochondria for ATP production and Ca^2+^ handling. Compartmentalized bioenergetic systems including the AIS, myelinated segments, and presynaptic boutons, exhibit distinct regulatory mechanisms and reliance on NAD⁺ homeostasis and glial metabolic support [49, 70]. Astrocytes further contribute to synaptic function through close structural coupling at axon-spine interfaces, where their coverage level modulates synaptic strength, calcium dynamics, and transmitter clearance; degradation of this glial interface leads to neurotransmitter spillover and synaptic dysfunction [71]. Synaptic strength across axonal arbors is intrinsically heterogeneous, exhibiting variability in short-term plasticity that is mediated by release probability determinants such as Unc13A, reflecting a functional trade-off between signaling reliability and energy cost [72, 73]. This functional architecture is dynamically regulated by experience-dependent and circadian factors, as demonstrated in the mouse retina, where visual cues and astrocytic phenotypes guide inhibitory synapse maturation and circuit refinement [74, 75].

#### Local axonal translation

Local protein synthesis within axons is fundamentally regulated by the precise subcellular localization of mRNAs and the assembly of translational complexes. This process is primarily directed by interactions between the 3' untranslated regions (3' UTRs) of mRNAs and specific RNA-binding proteins (RBPs), such as adenomatous polyposis coli (APC), which governs the transport and local translation of β-actin mRNA [68, 70, 76, 77]. The axonal endoplasmic reticulum provides a structural platform for this localized translation, ensuring spatiotemporal control over the axonal proteome during development [78]. Furthermore, the formation of specialized "translation factories" and the distribution of sparsely docked RNA granules enhance local translation efficiency and enable synapse-specific regulation, thereby supporting axonal growth and synaptic maturation [14, 79].

This intricate translational system is modulated by multiple regulatory layers. RBPs play pivotal roles: CPEB2 controls the translation of *VGLUT2* mRNA to support presynaptic long-term potentiation and memory [80]; hnRNP R is involved in the axonal localization and translation of *MAPT* mRNA [81, 82]; and FMRP regulates activity-dependent translation through the formation of phase-separated condensates [83]. RNA modifications provide another regulatory dimension: N6-methyladenosine (m^6^A) controls the local translation of APC via YTH domain-containing family (YTHDF) m^6^A reader YTHDF1. Mutations in the m^6^A writer METTL14 are linked to neurodevelopmental disorders. In addition, N4-acetylcytidine modifications enhance translational efficiency and mRNA stability [84]. Additionally, mechanistic target of rapamycin (mTOR) activation by presynaptic NMDA receptors, and microRNAs that differentially control the local translation of hundreds of transcripts, further refine this regulatory network to maintain neurotransmitter release pools and axonal responsiveness [79, 85].

Functionally, local translation is indispensable for synaptic plasticity, as demonstrated by the axonal location and translation of *Agrn* mRNA at the neuromuscular junction, which is essential for synapse transmission and motor function [86], and for maintaining the releasable vesicle pool in excitatory neurons [79, 80]. In axonal development and repair, injury-induced changes in RNA polyadenylation and RBP activity—such as activity of PTBP1 that regulates KPNB1 and RHOA translation—promote regenerative outgrowth, whereas deficiency in hnRNP R or PTBP2 impairs the synthesis of cytoskeletal proteins like β-actin, leading to growth defects and denervation [87–89]. This mechanism also underpins memory processes. In *Drosophila,* axonal mRNA localization and translation is regulated by RBPs like Imp and is essential for long-term behavioral memory consolidation. In rodent models, local axonal mRNA translation is essential for learning-induced presynaptic plasticity [80, 90]. In the mature nervous system, local translation increasingly supports energy metabolism, shifting towards the production of proteins involved in glucose catabolism to ensure synaptic maintenance and function at structures like the neuromuscular junction [86].

**Fig. 1:**
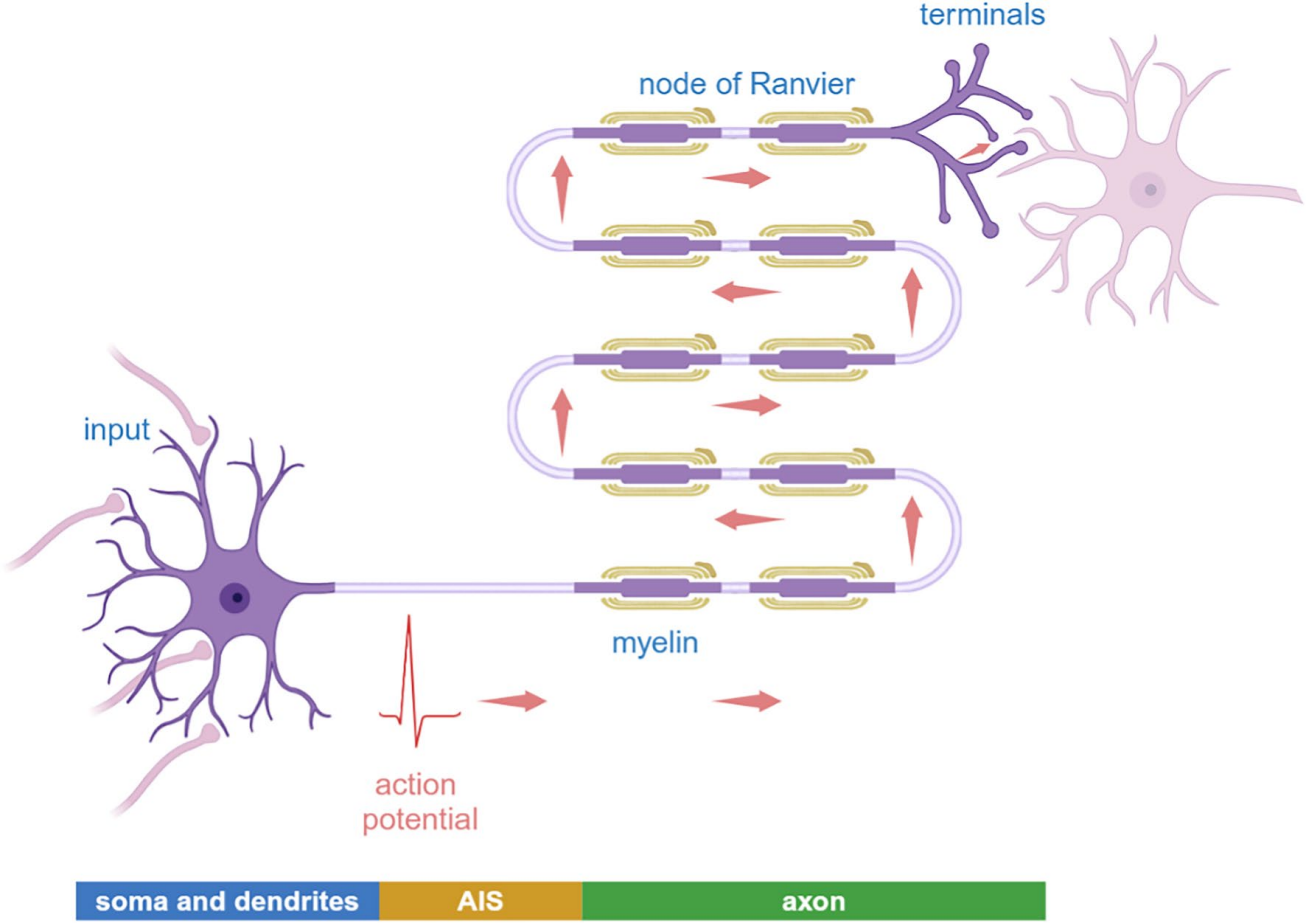
Schematic of a typical neuron. Neurons are structurally distinct from other cells. A typical mammalian neuron has a cell body (also known as the soma) with an average size of ~ 20 µm in diameter. The many highly branched processes projected from the soma are called dendrites whose function is to receive inputs from other cells. Neurons connect with their target(s) via long processes called axons that are in most cases enclosed in myelin sheaths separated by nodes of Ranvier. Each neuron typically has one axon that forms synapses with the target(s). Action potentials, initiated in the axonal initiation segment (AIS) proximal to the soma, can be propagated and transmitted down to the axons at a speed up to 90 m per second

In brief, axons carry out numerous tasks that are fundamental to neuronal function. Their sophisticated structures and extraordinary energy demand render them extremely vulnerable to damage by genetic factors, epigenetic factors, environmental stress, and aging (Fig. 1).

## Axonal dysfunction and neurodegenerative diseases

**Fig. 2:**
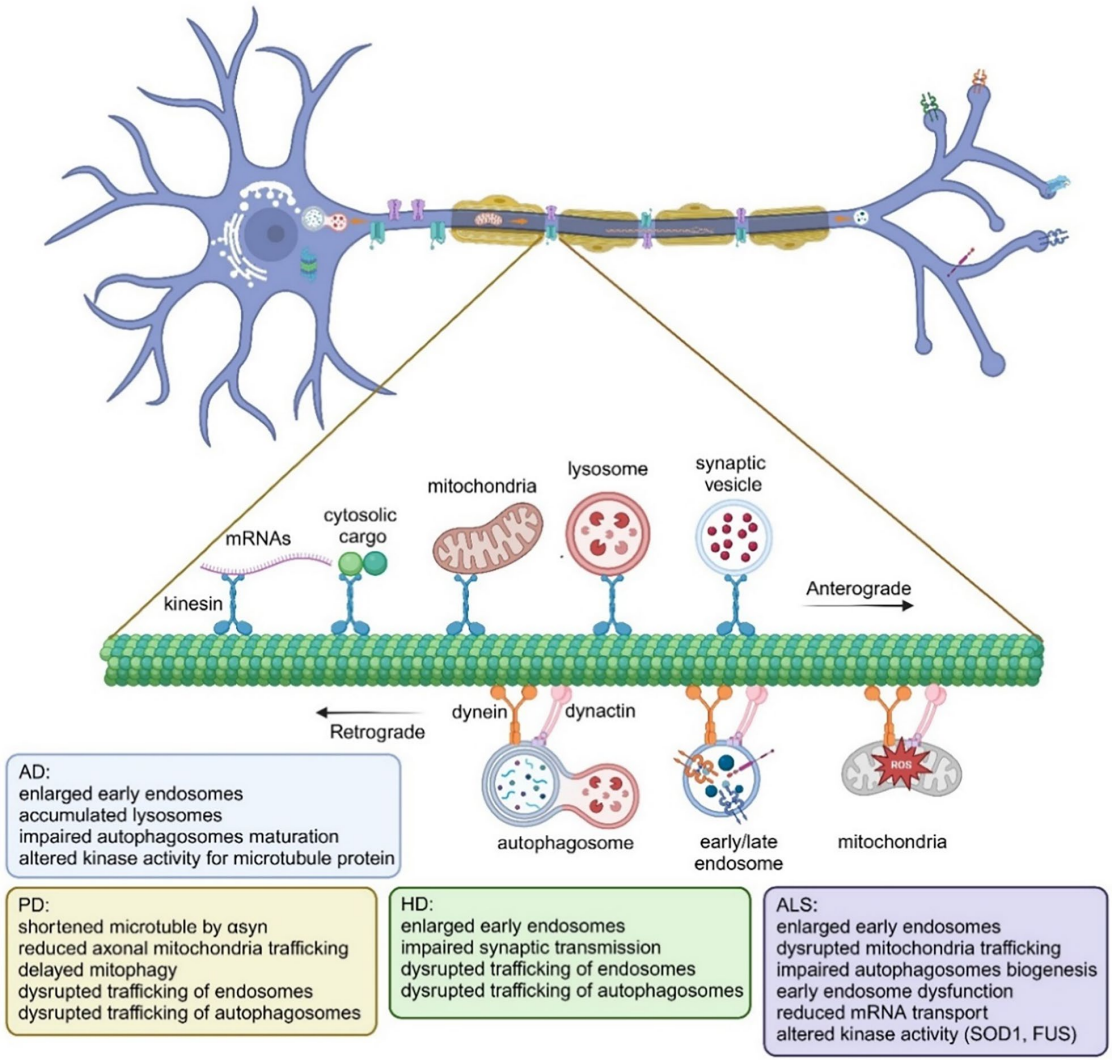
The many facts of axonal dysfunction in neurodegeneration. Axonal trafficking is a critical process for neuronal homeostasis ranging from endosomal-lysosomal pathways to mitochondrial turnovers. Alteration in any of these critical pathways can lead to eventual neurodegeneration. For the pathogenesis of different neurodegenerative diseases, they share some common pathways, and they also have some unique contributors

Given the unique and critical structural and functional roles of axons in neuronal development and maintenance, it is not surprising that changes in axonal structure and function are implicated in many neurodegenerative disorders such as AD, PD, and Huntington’s disease (HD). Axonopathy is a neurotoxic disorder with a variety of lesions in the axons of the brain, spinal cord, and peripheral nerves. It can involve structural or functional defects in the axon or its terminal, such as swelling, fragmentation, or loss. Axonopathy can also include alterations in the cytoskeleton and defects in axonal transport. Dysfunction or degeneration of axons (axonopathy), broadly defined as structural and functional defects in the axon or its terminal, has been established as an early and prominent contributing mechanism to the pathogenesis, progression, and symptomology of neurodegenerative disorders. Many excellent reviews have been recently published [34, 91]. The major pathways are summarized in Fig. 2. Below we only focus on early steps/events in endocytic trafficking that are impacted in neurodegenerative diseases.

### AD

#### Amyloid-β precursor protein (APP) processing and amyloid-β (Aβ) production

The pathological cleavage of APP into Aβ peptides is a cornerstone of AD pathogenesis. This amyloidogenic processing is initiated by the altered subcellular convergence of APP and beta-secretase 1 (BACE1) within endocytic compartments [92, 93]. Subsequent cleavage by γ-secretase generates Aβ peptides of varying lengths, with the shift toward longer, more toxic species being a critical determinant of pathogenicity [94, 95]. This process is spatially regulated, as increased axonal transport of APP leads to the presynaptic accumulation of its C-terminal fragments, resulting in increased local Aβ production [96]. This is further exacerbated by the pathological bidirectional axis between mitochondria and Aβ: mitochondrial-localized APP promotes Aβ production, while Aβ itself impairs mitochondrial respiration; conversely, mitochondrial dysfunction upregulates Aβ generation, creating a vicious cycle that disrupts energy-dependent processes like axonal transport [97]. This cascade is amplified by pro-degenerative signaling, where stimuli like proNGF can trigger APP synthesis in axons, directly contributing to axonal degeneration [25]. In response, therapeutic strategies have evolved to include not only direct BACE1 inhibition [98, 99] but also γ-secretase modulators that favor generation of shorter, less amyloidogenic Aβ fragments [100, 101] and approaches that enhance the non-amyloidogenic α-secretase pathway [102, 103]. Novel strategies are now exploiting subcellular APP redistribution by targeting NCAM2 (neural cell adhesion molecule 2 protein)–APP interaction [99] or the lactoferrin–Arf6–Rab11 axis [104], or enhancing Aβ clearance via degradation pathways [105, 106]. Genetic interventions, such as exon-skipping antisense oligonucleotides, offer another avenue by generating truncated APP isoforms lacking critical BACE1 cleavage sites [92]. The overall goal of these strategies is to decrease toxic Aβ accumulation by reducing APP/BACE1 co-localization in lipid rafts [107] or enhancing PINK1-mediated mitophagy [108], without disrupting the potential physiological functions of APP and Aβ. Clinical translation relies on advanced immunotherapies [109] and validated biomarkers for early intervention.

#### Roles of genetic risk factors in the impairment of axonal function

Derangement of axonal transport pathways [110] accompanied by early axonal structural and transport deficits is widespread in AD. Combined PET/MRI and animal studies revealed that degeneration of the basal forebrain cholinergic projection to the entorhinal cortex represents an early feature of cognitive aging. This degeneration is characterized by reduced VAChT (vesicular acetylcholine transporter) density in the entorhinal cortex, which correlates with impaired spatial object memory and is accelerated in Aβ^+^tau^+^ models [111]. Endocytic trafficking and axonal transport are predominantly governed by the small Rab GTPases [112–114]. Rab5 marks early endosomes. Early-endosomal dysregulation caused by Rab5 hyperactivation is linked to prodromal and neurodegenerative cellular phenotypes of AD [115, 116]. Upon activation by guanidine nucleotide exchange factors (GEFs), Rab5 is converted from GDP- to GTP-bound form and mediates endocytic trafficking. Then the GTP-bound form of Rab5 is returned to the inactivated state through deactivation by GTPase activating proteins (GAP). Prolonged activation of Rab5 impairs neuronal trafficking in AD [114].

Of the more than 70 genetic risk factors for late-onset AD (LOAD) that have been identified by recent large-scale genome-wide association studies (GWAS) [117–119], many are involved in the regulation of endocytic trafficking [120], including Ras and Rab Interactor 3 (RIN3). RIN3, along with two other risk factors Bridging Integrator 1 (BIN1) and CD2-associated protein (CD2AP), has been consistently picked up in six large GWAS thus far [120]. Consistent with RIN3 as an activator for Rab5, epigenetic studies have revealed that RIN3 expression is likely increased in early phase of AD pathogenesis [121, 122].

Our recent study has demonstrated that: 1) increased expression of RIN3 activates Rab5 and disrupts axonal trafficking and processing of APP; and 2) RIN3 recruits and forms a complex with CD2AP and BIN1 on Rab5-positive early endosomes [123]. One of the attractive hypotheses to explain the role of RIN3/BIN1/CD2AP in AD pathogenesis is that RIN3/CD2AP enhances the beta-cleavage of APP, eventually generating Aβ [124, 125], while RIN3/BIN1 promotes tau phosphorylation by GSK3β (glycogen synthase kinase 3 beta) [126, 127]. BIN1 may also act through RIN3 to contribute to the beta-cleavage of APP [128]. Direct targeting of Rab5 expression and activity represents a promising therapeutic strategy. Rab5-specific antisense oligonucleotides administered in the Dp16 Down syndrome mouse model effectively reduced Rab5 levels, normalized endosomal Rab activity and GEF (Rabaptin-5 and Rabex-5) recruitment, and reduced pathological hallmarks including tau hyperphosphorylation and axonal transport deficits, with good tolerability [129]. Expression of a dominant-negative Rab5 mutant (Rab5^S34N^), which is defective in GTP-binding, rescued the β-CTF (β-carboxyl terminal fragment)-induced basal forebrain cholinergic neuron (BFCN) atrophy and NGF signaling impairment in experimental models, validating Rab5 as a druggable target [130, 131]. Indirect pharmacological modulation via the γ-secretase modulator BPN15606 reduced Rab5 hyperactivation, normalized neurotrophin signaling, synaptic protein levels, and tau phosphorylation without altering the levels of total Aβ [132]. Taken together, dysregulation of Rab5-mediated endosomal trafficking plays a significant role in the early stage of AD. While targeting Rab5 indirectly has been explored as a therapeutic strategy to prevent BFCN degeneration in Lewy body dementia [133], the relevant clinical measures have not proven effective.

#### Role of AIS in tauopathy

In mature neurons, the AIS serves as a gatekeeper, confining the microtubule-associated protein tau to the axonal compartment under physiological conditions [134]. However, this structure is highly vulnerable in neurodegenerative diseases. In AD, the AIS undergoes significant structural and functional alterations, including impaired length and integrity, which disrupt action potential initiation and neuronal output [135, 136]. This vulnerability is exacerbated in the presence of excessive APP, which disrupts the AIS and depresses neuronal activity [137, 138]. Critically, the loss of the gatekeeping function of AIS results in the distribution of pathogenic tau species from the axon into the somatodendritic compartments [6, 139, 140], where they interfere with synaptic vesicle function, and synergize with the detrimental effects of Aβ on synaptic structure, culminating in the learning and memory deficits characteristic of AD [139, 140]. The underlying mechanisms involve tau pathology impairing activity-dependent AIS plasticity, partly through downregulation of the master scaffolding protein Ankyrin G and disruption of local signaling pathways, such as SSTR3-ciliary-CREB, ultimately leading to altered distribution of voltage-gated channels and neuronal hyperexcitability [13, 141, 142].

AIS dysfunction and tau mislocalization are receiving increasing attention as therapeutic targets for AD. One approach involves stabilizing the structural integrity of the AIS itself. Pharmacological stabilization of microtubules has been shown to preserve AIS barrier function, prevent the tau hyperphosphorylation-induced AIS displacement, and rescue neuronal excitability [143]. Direct targeting of tau pathology includes isoform-specific targeting of 1N4R-tau, modulating tau phosphorylation kinetics and boosting its proteasomal clearance. Deubiquitinases like USP10 (ubiquitin specific peptidase 10) are emerging as potential targets to disrupt aggregation pathways [136, 144, 145]. Neuroprotective approaches such as sirtuin 1 (SIRT1) activation, can improve synaptic plasticity and reduce phospho-tau levels, thereby indirectly supporting AIS-related excitability [146]. Finally, restoring physiological AIS plasticity, for instance via cytoskeletal stabilizers or Kv7 channel agonists, counteracts pathological AIS changes and neuronal hyperexcitability, highlighting druggable mechanisms for maintaining this critical cellular gateway in tauopathies [147].

### HD

The pathogenesis of HD is characterized by multifaceted axonal dysfunction caused by the mutant huntingtin protein (mHTT). mHTT preferentially forms aggregates within axons, depleting cytosolic levels and concurrently inducing severe mitochondrial dysfunction, which is manifested as impaired energy metabolism and elevated reactive oxygen species (ROS) [148, 149]. This axonal accumulation disrupts mitochondrial integrity, compromises oxidative phosphorylation, and activates the SARM1-dependent axon degeneration pathway, a mechanism shared by other conditions like Charcot-Marie-Tooth disease type 2A [150]. mHTT also impairs axonal transport due to its disrupted scaffolding function, leading to deficient trafficking of essential cargoes, including mitochondria and neurotrophic factors [151]. Furthermore, mHTT disrupts the actin cytoskeleton in growth cones, thereby hindering axonal development and regeneration [151].

A central consequence of this transport disruption is the failure of cortico-striatal circuitry, a cardinal feature of HD [152]. The survival of striatal medium spiny neurons (MSNs) depends on a continuous supply of brain-derived neurotrophic factor (BDNF) from cortical afferents. mHTT undermines this trophic support by both repressing BDNF transcription in cortical neurons and impairing the anterograde axonal transport of BDNF to the striatum [153]. This results in region-specific pathology, with prominent degeneration of striatal MSN projections due to this trophic deficit, compounded by excitotoxicity and mitochondrial damage [154].

The axonal transport deficit extends to retrograde signaling. Wild-type HTT, in a complex with HAP40, binds to Rab5-positive early endosomes and is essential for retrograde transport of activated BDNF/TrkB signals [155]. mHTT disrupts this process by co-aggregating with and depleting functional wild-type HTT from Rab5 endosomes, thereby stalling retrograde signaling and leading to synaptic deficits [155, 156].

Strategies have been developed based on these findings, such as targeting the polyproline domain of mHTT to reduce its intrinsic toxicity [87, 157], and employing antioxidants or mitochondrial fission inhibitors to alleviate ROS-mediated damage [158]. A major focus has been put on restoring BDNF/TrkB signaling, which can be achieved by targeting Rab5 adaptor proteins like Hook1 (hook family of coiled-coil protein 1) or AP-2 (adaptor protein 2), or by promoting HTT phosphorylation to normalize BDNF transport dynamics [159, 160]. Pharmacological agents such as ginsenosides offer neuroprotection by activating BDNF/TrkB pathways, potentially via these Rab5-dependent mechanisms [161]. Concurrently, the role of Rab5 in autophagy provides a complementary strategy; inducing autophagy enhances the clearance of mHTT aggregates, and several compounds have demonstrated efficacy in reducing toxic mHTT levels and rescuing disease phenotypes [158, 161]. Finally, harnessing pro-regenerative signals from astrocyte-derived exosomes while counteracting the inhibitory effects of ApoE, represents another innovative approach to promote repair [162, 163].

### PD

#### α-Syn and retrograde axonal transport

Accumulating evidence indicates that the retrograde axonal transport of pathological α-syn is a pivotal mechanism in PD. Phosphorylated α-syn (pSer129) is enriched in cortical glutamatergic synapses in the putamen at early disease stages, preceding the formation of Lewy bodies in cortical regions, supporting retrograde transport of α-syn from distal synapses towards the soma [164]. This has been experimentally validated using the α-syn-linker-mKO2 fluorescent reporter and a bimolecular fluorescence complementation system, demonstrating that α-syn can undergo retrograde transport from the striatum to midbrain dopamine neurons, where it forms phosphorylated, detergent-resistant aggregates that evolve into Lewy body-like inclusions [164, 165]. Furthermore, microfluidic platform studies confirmed that pathological α-syn aggregates propagate between connected neurons via axonal transport, templating endogenous α-syn to misfold [166].

The pathological impact of α-syn is twofold: it not only propagates itself, but also disrupts essential axonal transport systems. In transgenic PD models overexpressing human α-syn, significant elevations in the levels of activated Rab5 and Rab7 correlate with impairment of retrograde axonal trafficking of BDNF in cortical neurons [167]. This suggests that either reducing α-syn expression or normalizing Rab5 activation could theoretically prevent these axonal transport defects. The molecular underpinnings of transport deficits also involve dysregulation of vesicular release, where aberrant α-syn interacts with Synaptotagmin-13 to disrupt extracellular vesicle dynamics [168].

Targeting these interconnected pathways offers promising therapeutic strategies. Passive immunotherapy using antibodies for C-terminal α-syn reduces intracellular α-syn accumulation, blocks pathological axonal transport, and improves neurological deficits in transgenic models [169]. The molecular tweezer CLR01 inhibits α-syn oligomerization in dopaminergic neurons, thereby preserving axonal transport function [170]. To address Rab5 hyperactivation, which disrupts endo-lysosomal trafficking, modulation of cholesterol homeostasis corrects the aberrant membrane localization of Rab5 effectors, effectively rescuing axonal transport [171]. Other approaches include interrupting α-syn retrograde spread, thereby blocking gut-to-brain transmission via the vagus nerve [172], or enhancing clearance of extracellular α-syn through astrocytic phagocytic pathways [173, 174]. Together, these interventions highlight the therapeutic potential of normalizing axonal transport dynamics in PD.

#### Transmission of α-syn pre-formed fibrils (PFF)

α-Syn PFFs act as pathogenic seeds that, upon neuronal uptake, can template endogenous α-syn to misfold and aggregate into Lewy body-like inclusions, thereby propagating pathology and driving neurodegeneration [175, 176]. A key mechanism for this uptake involves the specific, high-affinity (dissociation constant = 77 nM) binding of α-syn PFFs to lymphocyte-activation gene 3 (LAG3) [177]. LAG3 colocalizes with the endosomal GTPases Rab5 and Rab7, and its deletion significantly reduces PFF endocytosis, delays dopaminergic neuron loss, and ameliorates biochemical and behavioral deficits in vivo [177]. Beyond LAG3-mediated endocytosis, neuronal internalization of PFFs is also mediated by voltage-gated calcium channels and macropinocytosis, regulated by the Ca^2^^+^–Calmodulin–Calcineurin signaling pathway [178]. The structural polymorphisms of PFFs, such as the highly seeding-competent "mini-P" type versus the less active "mini-S" type, further influence their propagation efficiency [179]. PFFs can spread from the periphery to the central nervous system (CNS) via distinct routes, including the vagus nerve after gastrointestinal inoculation [172, 180] and the lymphatic system [181]. At the cellular level, microglia clear α-syn fibrils via pathways like the NLRP3 inflammasomes, and microglial dysfunction can exacerbate the pathological spread [173, 182].

The resulting pathology includes early synaptic dysfunction in cortico-striatal circuits and dysregulation of axonal guidance molecules like Slit2/Robo1, preceding significant neuronal loss [183, 184]. Furthermore, PFFs can activate the NLRP3 inflammasome, triggering a self-perpetuating cycle of neuroinflammation and neuronal injury [185].

Consequently, therapeutic strategies are focusing on inhibiting PFF assembly, spread, and uptake. LAG3 has emerged as a prominent target. Anti-LAG3 antibodies inhibit α-syn PFF internalization by disrupting amyloid precursor-like protein 1 (APLP1)–LAG3 interactions, effectively halting neurodegeneration in vivo and reducing gut-to-brain transmission [186, 187]. Synergistic targeting of the APLP1–LAG3 complex has demonstrated superior efficacy compared to LAG3 inhibition alone [187]. Small-molecule inhibitors like the histone deacetylase inhibitor Givinostat, can directly block α-syn C-terminal interactions with LAG3/RAGE receptors, suppressing the neuroinflammatory spread [188]. Other approaches include small molecules (e.g., CNS-11, CNS-11 g) that disassemble α-syn fibrils [189], antibody-based therapies that block neuronal uptake [190], and vaccines designed against α-syn fibril structures [191].

However, key challenges and controversies remain. Questions persist regarding neuronal LAG3 expression, as some studies show that LAG3 overexpression did not exacerbate pathology, and its knockout did not mitigate α-syn propagation [192]. Additional limitations include the lack of standardization of PFF preparation protocols [193, 194], potential differences in the propagation efficiency between rodent and human α-syn [195], and the unresolved question of whether peripheral organs can spontaneously generate α-syn pathology independently [196].

#### Parkin and clearance of damaged mitochondria

The E3 ubiquitin ligase Parkin and the serine/threonine kinase PINK1 constitute a core pathway for eliminating damaged mitochondria. Upon mitochondrial damage, PINK1 accumulates on the outer mitochondrial membrane where it phosphorylates both ubiquitin and Parkin's ubiquitin-like domain, leading to Parkin activation [61]. The activated, phosphorylated Parkin then ubiquitinates outer mitochondrial membrane proteins, marking the damaged organelle for clearance by recruiting autophagy receptors like OPTN and NDP52, thereby initiating mitophagy [197, 198]. Loss-of-function mutations in *PRKN* (encoding Parkin) and *PINK1* are common genetic causes of early-onset PD, resulting in defective mitophagy and accumulation of damaged mitochondria within neurons [199–201]. Dopaminergic neurons are particularly vulnerable to this dysfunction due to their high energetic demands and oxidative stress. Parkin deficiency leads to the buildup of impaired organelles, increased ROS, energy failure, and ultimately neuronal apoptosis in the substantia nigra [202]. The failure to clear damaged mitochondria exacerbates mitochondrial dysfunction, including loss of membrane potential, calcium dyshomeostasis, and impaired respiratory chain function, which amplifies neuronal injury through the release of pro-apoptotic factors and inflammatory signals [203].

Therapeutically, compounds like β-NMN and ginsenoside Rb1 can restore mitophagy and alleviate toxicity [203, 204]. Furthermore, regulators like Rab11, and endoplasmic reticulum–mitochondria contact sites as critical structures for cellular function, fine-tune Parkin-mediated mitophagy [205]. TRIM5α enhances the autophagic efficiency by assembling mitophagy machinery with TBK1 and autophagy adaptors [206]. However, controversies remain that highlight the complexity of mitophagy. For example, a study reported a non-mitophagic, neuronal activity-dependent neuroprotective role of Parkin via mechanisms such as CaMK2-dependent phosphorylation [207]. The significance of this alternative function, particularly in contrasting basal and stress conditions, is a subject of active debate [208].

#### Leucine-rich repeat kinase 2 (LRRK2) and endocytic trafficking

LRRK2, a member of the ROCO protein family, is a multidomain serine-threonine kinase that regulates crucial cellular processes including endolysosomal trafficking, vesicle transport, autophagy, and mitochondrial function [209]. Mutations in *LRRK2* represent the most common genetic causes of familial PD and a significant risk factor for sporadic cases [210–212]. Pathogenic mutations, such as G2019S, lead to hyperactivation of its kinase activity [210, 211], which in turn phosphorylates specific Rab GTPases (e.g., Rab10, Rab12). Pathogenic *LRRK2* variants sustain the activation of Rab5, which may be a critical first step in PD pathogenesis by altering early endocytic pathways [209]. The aberrant phosphorylation of Rabs disrupts their interaction with effector proteins, impairing the dynamic balance of endocytic transport, vesicle formation, receptor recycling, and lysosomal function [213, 214].

The consequent disruption of the endolysosomal pathway manifests in key PD pathologies. LRRK2 hyperactivation promotes neuronal internalization of α-syn PFFs, fostering the formation of toxic intracellular aggregates [215]. It also disrupts synaptic vesicle recycling, leading to the accumulation of toxic oxidized dopamine and the selective vulnerability of midbrain dopaminergic neurons [216, 217]. Furthermore, LRRK2 is recruited to and activated by damaged lysosomes, creating a feed-forward loop that exacerbates lysosomal homeostasis imbalance and compromises cellular waste clearance [218, 219].

Given that elevated LRRK2 kinase activity is a key disease driver, targeting the LRRK2–Rab phosphorylation axis may be a promising therapeutic strategy. The primary focus has been on the development of LRRK2 kinase inhibitors, several of which are currently in clinical trials [210, 220]. These approaches are complemented by strategies that restore Rab homeostasis, such as promoting Rab dephosphorylation through modulation of the PPM1H phosphatase [221] and disrupting pathogenic feedback loops using inhibitors of Rab-LRRK2 interactions [222]. Preclinical studies demonstrate that LRRK2 inhibition can restore endocytic transport, alleviate vesicle trafficking deficits, and reduce α-syn accumulation and mitochondrial damage [210, 211, 223]. For instance, the novel inhibitor FL090 ameliorates pathological phenotypes by reducing the kinase activity [223]. Additionally, modulating the recruitment of LRRK2 to lysosomes via systems like ATG8 presents another potential intervention [218]. Collectively, these interventions aim to normalize endosomal trafficking and neuronal function, particularly in patients with *LRRK2* mutations or increased Rab phosphorylation levels. The pathogenic role of LRRK2 is further underscored by its functional crosstalk with other PD-linked proteins. This interplay is exemplified by the finding that LRRK2 can impair endophilin-A-mediated endocytosis—a pathway also disrupted in the absence of Parkin—leading to synaptic vesicle recycling defects [217]. Thus, LRRK2 likely acts in concert with other PD-related proteins by disrupting shared endocytic pathways to drive neurodegeneration [224].

### Amyotrophic lateral sclerosis (ALS)

The pathogenesis of ALS is closely linked to dysfunction in endosomal trafficking, particularly through the loss-of-function of key genes such as *C9orf72* and *ALS2* (Alsin Rho guanine nucleotide exchange factor 2). The absence of C9orf72, a Rab GEF, primarily disrupts endosomal maturation rather than directly inducing canonical autophagic phenotypes. In C9orf72-knockout motor neurons derived from induced pluripotent stem cells (iPSCs), this disruption manifests as defective endosomal maturation, concomitant with lysosomal homeostasis impairment and inhibition of autophagic flux [225]. The loss of C9orf72 reduces Rab5 activation, compromising early endosome function and vesicular trafficking [225, 226]. This deficit is further exacerbated by decreased SEC16A protein level and mislocalization of SEC12 at endoplasmic reticulum exit sites, preventing proper SAR1 association and thereby disrupting endosomal transport [226]. Additionally, C9orf72 deficiency aggravates the transactive response DNA binding protein of 43 kDa (TDP-43)-mediated neurotoxicity by impairing autophagic clearance [227]. C9orf72 ALS patient-derived motor neurons show increased TBK1 phosphorylation at S172, suggesting that the autophagic-lysosomal dysregulation is primarily driven by toxic gain-of-function mechanisms [225].

Parallel deficits are observed with *ALS2* mutations. ALS2, another Rab5 GEF, is critical for synaptic development; its loss reduces Rab5 activation, disrupts endosomal trafficking and sorting, and impairs synaptic vesicle release [228, 229]. This mechanism is similar as C9orf72 mutation, as in zebrafish models C9orf72 regulates presynaptic vesicle transport and release to maintain neuromuscular junction (NMJ) integrity, and its loss directly impairs synaptic transmission, contributing to ALS/frontotemporal dementia (FTD)-related neuromuscular dysfunction [229].

Other ALS-associated genes are implicated in the disruption of the endolysosomal network. For instance, mutation(s) in NEK1, which phosphorylates VPS26B to regulate retromer-mediated endosomal trafficking, disrupt plasma membrane protein transport and induce mitochondrial and lysosomal dysfunction upon loss [230]. Moreover, multi-gene analyses reveal that risk genes (e.g., *GGNBP2*, *ATXN3*, *SLC9A8*) are enriched in pathways governing membrane and vesicle-mediated transport, indicating that a synergistic disruption of the endolysosomal network constitutes a key genetic basis for ALS [231].

Therapeutically, targeting these pathways holds significant promise. Studies indicate that expressing constitutively active Rab5 or restoring C9orf72 levels can reverse endosomal trafficking defects and associated neurodegeneration [226, 232], offering novel strategic avenues for ALS.

## Impacts of glia on axonal function in health and disease

**Fig. 3:**
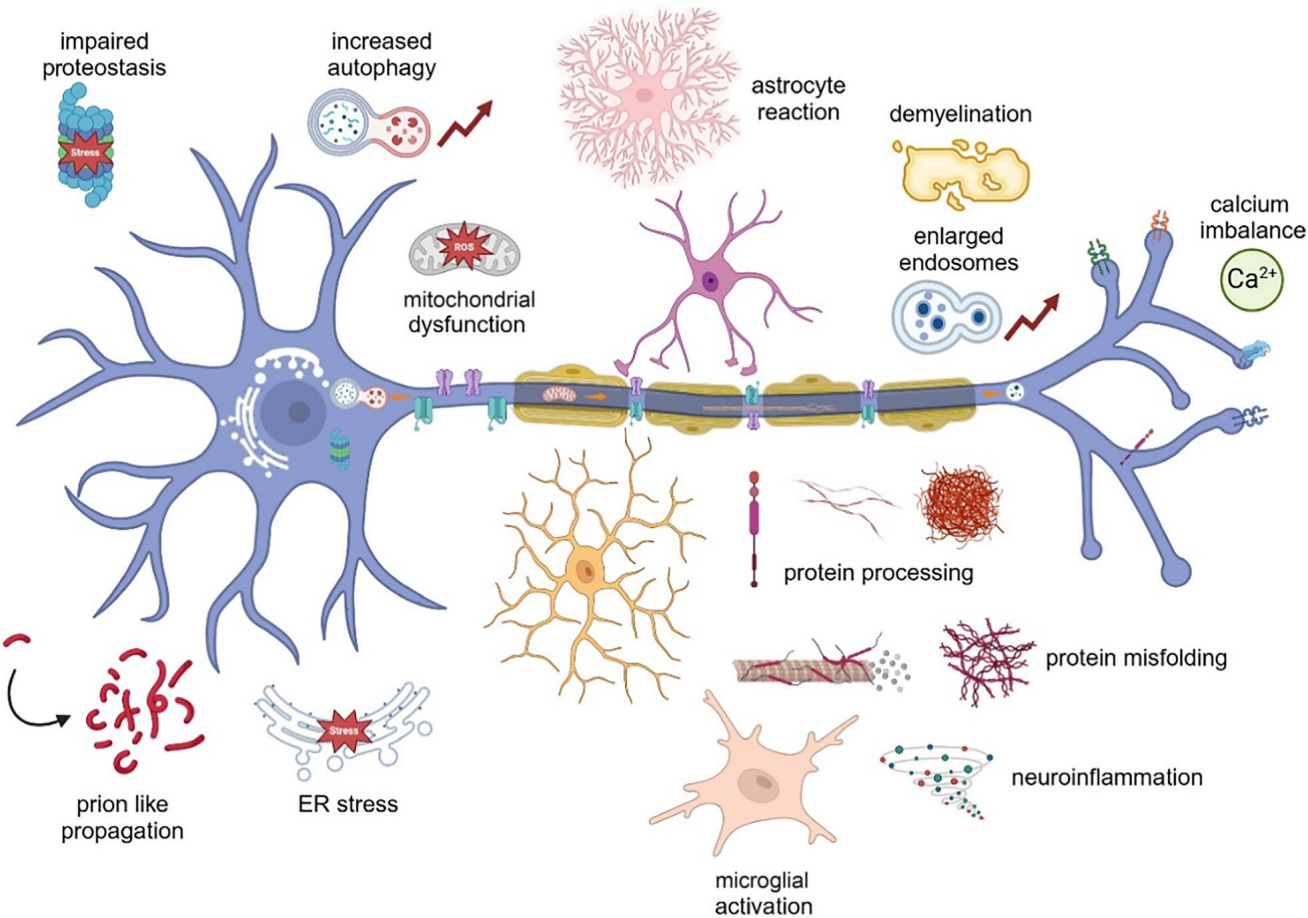
A comprehensive overview of neurodegeneration. The cellular processes that are impacted in neurodegeneration as depicted in Fig. 2 can also be directly or indirectly impacted by astrocytes and microglia. Pathological conditions such as genetic mutations, oxidative stress, etc., can activate astrocytes and microglia of the brain to initiate the process of neurodegeneration

Although not the focus of this review, we fully recognize that glia can play important roles in axonal structure and function. Under pathogenic conditions, they may play a pivital role in the onset and exacerbation of neurodegenerative processes through multiple mechanisms, including their effects on axons (Fig. 3).

### Physiological regulation of axonal homeostasis by astrocytes and microglia

#### *Metabolic* s*upport*

The lactate shuttle mechanism, fundamentally reliant on monocarboxylate transporters (MCTs), constitutes a critical pathway for supplying metabolic substrates to axons. Within the astrocyte-neuron lactate shuttle, astrocytes metabolize glucose to lactate, which is subsequently exported via MCTs (predominantly MCT1 and MCT4) and imported into neurons primarily through MCT2. Neurons then convert lactate to pyruvate for oxidative phosphorylation in the tricarboxylic acid cycle, generating ATP essential for synaptic transmission, axonal function, and processes such as memory formation [233–235]. Oligodendrocytes similarly provide metabolic support to axons by shuttling lactate and other metabolites via MCTs; dysfunction in this pathway disrupts axonal integrity and contributes to neurodegeneration [236, 237]. The cell-specific expression of MCT isoforms is crucial: MCT2 facilitates neuronal lactate uptake [233, 238], MCT4 mediates astrocytic lactate release [239], while MCT1, expressed on astrocytes and endothelial cells (ECs), regulates broader lactate flux [240]. Lactate dehydrogenase (LDH), particularly the LDHA and LDHB isoforms, dynamically regulates the interconversion of lactate and pyruvate; impaired LDH activity within axons disrupts lactate utilization [52, 237]. The efficiency of lactate shuttling is further modulated by specific regulators, such as SETDB1-dependent post-translational modifications governing MCT1 stability [240] and MCT4-mediated lactate supply sustaining orexin neuronal activity and arousal [239]. Axonal regeneration following injury has a high energy demand, met by local glycolysis, sustained mitochondrial transport, and lactate imported via MCTs (e.g., MCT2) [30, 241, 242]. Consequently, inhibiting lactate production or transport significantly impairs the regenerative capacity [30, 243]. Metabolic adaptations, including overcoming lactate shuttle deficiencies (e.g., by enhancing EC MCT1 after spinal cord injury) or modulating lactate metabolism via regulators like Pdhb (acting through MCT2), are thus vital for restoring neuronal energy balance and facilitating axonal regeneration and functional recovery [243].

#### Glutamate homeostasis

Maintaining extracellular glutamate homeostasis is paramount for preventing excitotoxic neuronal death. This process is predominantly mediated by astrocytic excitatory amino acid transporters (EAATs). EAAT2 (GLT-1), the principal glutamate transporter on astrocytes, clears > 80% of synaptic glutamate, ensuring low extracellular concentrations. Its dysfunction leads to glutamate accumulation and excitotoxicity, which is critically implicated in AD (contributing to tau-mediated neurodegeneration) [244], HD [245], and traumatic brain injury (TBI). EAAT1 cooperatively supports glutamate clearance in select neuronal populations, including human iPSC-derived neurons, where its expression correlates with glutamate tolerance, though the precise mechanisms warrant further investigation [246]. Pathological downregulation of these transporters occurs across diverse contexts. In TBI, impaired EAAT2 clearance exacerbates excitotoxicity and neurological deficits [247]; in obesity, high-fat diets suppress astrocytic glutamate transporter expression in the brainstem, potentially elevating the cardiovascular risk via excitotoxicity [248]; and during retinal aging, the altered glutamate/GABA transporter expression disrupts the inhibitory-excitatory balance, accelerating neurodegeneration [249, 250]. The inhibitory GABAergic system can counteract the excitotoxicity, where GABA transporter (GAT) activity crucially regulates neuronal excitability. Reduced GABAergic tone and upregulated GATs post-spinal cord injury disrupt the excitation-inhibition balance, promoting spasticity and neuropathic pain, and exacerbating excitotoxicity [250, 251]. Furthermore, enhanced GAT activity facilitates glutamate recycling via the GABA/glutamate/glutamine cycle, indirectly supporting glutamate homeostasis, while strategies augmenting GABA synthesis or transport (e.g., probiotics) demonstrate neuroprotective potential by restoring GABA levels [252, 253]. Astrocytes are central to these protective mechanisms, primarily via EAAT2-mediated glutamate clearance [247]. Under excitotoxic stress, they may upregulate glutamate transporters and glutamine synthetase to accelerate glutamate detoxification [254]. This role is compromised in AD by Aβ42-induced EAAT2 impairment, leading to synaptic dysfunction. Compensatory upregulation of astrocytic glutamate transporters and neuronal GABA receptors in HD models may delay the excitotoxic damage [255, 256], highlighting endogenous neuroprotective adaptations. Pharmacological targeting of transporters offers therapeutic promise. One example is the EAAT2 positive allosteric modulators (e.g., GT951) that enhance transport activity and confer neuroprotection [257].

#### ROS clearance

Astrocytes play a central role in safeguarding neuronal mitochondria, particularly those in axons, from oxidative damage through multifaceted antioxidant mechanisms. Primarily, astrocytes serve as the brain's main producers of glutathione (GSH), synthesizing and exporting either GSH itself or its precursors (e.g., cysteine) for neuronal uptake and subsequent GSH synthesis [258]. This neuronally synthesized GSH is a critical antioxidant that neutralizes ROS, such as hydrogen peroxide, thereby protecting mitochondrial integrity [258]. The transcription factor nuclear factor erythroid 2-related factor 2 (Nrf2) orchestrates this process within astrocytes, upregulating GSH-synthesizing enzymes upon activation to enhance the ROS clearance capacity [259]. Furthermore, astrocyte-mediated GSH release modulates the extracellular redox environment, notably by reducing the cysteine/cystine ratio, which promotes neuronal cysteine uptake and sustains neuronal GSH pools, indirectly shielding mitochondria—the primary site of ROS generation—from dysfunction [259]. Beyond molecular transfer, astrocytes directly protect neurons by transferring functional mitochondria to neurons under pathological conditions (e.g., oxidative stress, neurotoxin exposure). These donated mitochondria integrate into recipient neurons, restore cellular viability, repair ROS-induced damage (including reversal of hydrogen peroxide injury), and provide essential metabolic support ("energy repair") to axonal mitochondria [260, 261]. This transfer constitutes a dynamic ROS clearance mechanism, as astrocyte-derived mitochondria not only supply ATP but also actively lower neuronal ROS levels and reverse neurotoxicity, preserving mitochondrial integrity in axons and soma. Astrocytes also coordinate a broader antioxidant enzyme network, regulating enzymes such as superoxide dismutase (SOD), glutathione peroxidase, peroxiredoxin-6 (PRX6), and glutathione reductase. This network acts synergistically to maintain redox homeostasis in the mitochondrial environment, as evidenced by PRX6-mediated neuroprotection in ischemia–reperfusion and the cooperative ROS scavenging by SOD and glutathione reductase [262]. The GSH/precursor provision, functional mitochondrial transfer, and regulation of antioxidant enzymes (including the Nrf2 pathway, PRX6, and SOD), collectively create a supportive, antioxidant microenvironment for neurons [263]. While physiological levels of astrocytic mitochondrial ROS (mROS) may act as signaling molecules, during disease states, their robust clearance systems are activated to provide critical, multi-layered protection for neuronal mitochondria against oxidative insults.

#### Nutritional support

Astrocytes function as pivotal supporting cells within the CNS by secreting key neurotrophic factors, including BDNF [264, 265], glial cell line-derived neurotrophic factor (GDNF) [266], and NGF [267], which regulate neuronal development, axonal growth, synaptic plasticity, and neural maintenance. Loss of astrocytic support precipitates synaptic degeneration, neuronal death, and broader neurodegenerative pathology. BDNF directly promotes axonal regeneration and reconnection post-injury [268] and sustains synaptic stability through the TrkB-mediated enhancement of synaptic strength and long-term plasticity, which is essential for memory formation and network maintenance. Conversely, deficient BDNF secretion contributes to synaptic dysfunction in neurodegeneration [269]. GDNF release, primarily regulated by the MAPK1/2 pathway (enhanced upon Kir4.1 potassium channel knockout) [270], robustly drives axonal regrowth and functional reinnervation in neural injury models, while also contributing to synaptic preservation and neural circuit maintenance [271]. Insulin growth factor 1 (IGF-1) secretion from astrocytes, upregulated by neural activity and modulated by factors like exercise and probiotic interventions [272], indirectly supports axonal growth via pro-survival effects and directly enhances synaptic plasticity, memory consolidation, and network remodeling through MAPK signaling. Astrocytic IGF-1 overexpression protects against axonal damage and demyelination [273] and improves synaptic strength during recovery.

#### Microglial surveillance and synaptic pruning

The C1q–C3–CR3 complement cascade constitutes a fundamental molecular pathway for synaptic pruning, crucially shaping neural circuitry during development and in response to injury or pathology. This process initiates with the deposition of the recognition molecule C1q onto postsynaptic membranes, such as neuronal dendrites, triggered by neuronal stress, inflammation, or developmental cues. C1q expression increases with age in regions like the medial nucleus of the trapezoid body, facilitating synaptic remodeling [274]. Activated C1q drives the local conversion and excessive deposition of complement component C3 at synaptic sites. C3 acts as a central opsonin, with its cleavage products (including C3a) directly tagging synapses for elimination [275]. Microglia, the primary phagocytes of the CNS, then recognize these deposited C3 fragments via the complement receptor CR3 (CD11b/CD18), initiating phagocytosis and the physical removal of tagged synaptic elements or axon terminals [276]. When microglial function is compromised, astrocytes provide compensatory debris clearance through C4b-mediated opsonization. Engulfed axonal fragments are subsequently degraded within astrocytes via the RUBICON-dependent LC3-associated phagocytosis pathway [277].

Cellular coordination underpins synaptic homeostasis. Microglia utilize CR3 for synaptic phagocytosis but exhibit "turf competition" that limits their capacity to clear their own cellular debris [277]. Consequently, microglial remnants are predominantly cleared by astrocytes via the C4b opsonization route [277]. Vascular endothelial cells further modulate this system by releasing CCL17, which activates astrocytic CCR4 receptors to promote C3 production and indirectly regulate microglial phagocytosis [278]. Key regulatory factors safeguard against aberrant pruning: CD55 (a complement inhibitor) suppresses cascade overactivation to protect synapses from pathological elimination [279]; triggering receptor expressed on myeloid cells 2 protein (TREM2) maintains microglial phagocytic tolerance by modulating complement gene expression [280]; and C1q-neutralizing antibodies block initiation of the cascade, reducing CR3-mediated phagocytosis and preserving synaptic integrity [281, 282].

### Glial dysfunction as a driver of axonopathy in neurodegenerative diseases

#### Oligodendrocyte pathology & demyelination-induced axonopathy

Oligodendrocyte dysfunction represents a central pathological mechanism in neurodegenerative diseases, directly driving demyelination and subsequent axonal degeneration. As the primary myelinating cells of the CNS, oligodendrocytes ensheath axons to facilitate rapid saltatory conduction while providing critical metabolic support and structural protection [283]. Their impairment or loss disrupts myelin integrity, leading to demyelination and axonal vulnerability [284]. In AD, Aβ oligomer toxicity induces oligodendrocyte damage, characterized by reduced MBP (myelin basic protein) expression, myelin degeneration, and white matter pathology, which collectively drive cognitive decline [285, 286]. ALS features cytoplasmic TDP-43 accumulation within oligodendrocytes, causing myelin disruption and initiating axonopathy, evidenced by axonal spheroid formation [287]. Multiple sclerosis involves autoimmune-mediated oligodendrocyte destruction, resulting in recurrent demyelination and progressive axonal loss [288]. Demyelination itself triggers axonal pathology through multiple mechanisms. Exposure of ion channels causes conduction failure and energy imbalance, precipitating axonal degeneration (axonopathy) [284, 289]. Demyelination-induced energy crisis is further exacerbated by a direct loss of intracellular ATP, which increases axoplasmic viscosity and drives the pathological liquid-phase separation and aggregation of proteins like TDP-43 and α-syn. This process is mechanistically distinct and can be rescued by restoring NAD^+^ metabolism, which normalizes ATP levels and reduces protein aggregation, highlighting a direct link between axonal bioenergetics and proteostasis [54]. This energy crisis and resulting protein aggregation cascade manifest as neurofilament disorganization, focal axolemmal rupture [290], and spheroid accumulation (prominent in ALS/TDP-43 models) [291, 292], culminating in chronic neurodegeneration [288]. Demyelination also activates microglia and astrocytes, releasing pro-inflammatory cytokines (e.g., tumor necrosis factor alpha [TNF-α], interleukin-1 beta [IL-1β]) that exacerbate axonal injury [49]. Furthermore, Aβ-induced oligodendrocyte pathology promotes neuronal damage via oxidative stress and ferroptosis [293]. Molecular interventions include normalizing NAD^+^ metabolism or mitigating oxidative stress to alleviate axonal energy crisis [49], alongside direct targeting of pathogenic oligodendrocyte processes.

#### Reactive astrocytosis and axonal degeneration

Reactive astrogliosis is a reaction of astrocytes in which astrocytes transit from a homeostatic supportive state (e.g., nutrient provision, ion buffering) towards a neurotoxic state. Reactive astrogliosis is prevalent in AD, PD, and ALS [294, 295], and involves morphological, transcriptomic, and functional remodeling that actively drives neuronal death [296, 297]. Pathologically, reactive astrogliosis correlates with Aβ plaques, tau hyperphosphorylation cascades, synaptic dysfunction, and neuroinflammation through inflammatory mediators [298]. The gut microbiota metabolite propionate attenuates astrogliosis and Aβ deposition by suppressing peripheral Th17 cells and IL-17 release, highlighting inflammatory pathways as key therapeutic targets [299]. Single-cell analysis further revealed significant astrocyte heterogeneity [300], with astrocytes in distinct disease stages or regions exhibiting either pro-inflammatory or protective phenotypes [301, 302]. Modulating astrocyte reactivity holds therapeutic promise. For example, HDAC (histone deacetylase) inhibition restored autophagic-lysosomal function and mitigated tau-induced astrocyte toxicity [303]. Precision strategies targeting this molecular diversity are under investigation.

Axonal degeneration emerges as an early event preceding neuronal apoptosis in multiple sclerosis, ALS, and AD [304, 305]. Critically, axonal damage and reactive astrogliosis interact reciprocally: axonal injury induces local astrogliosis [306] (e.g., traumatic brain injury accelerating AD/PD neurodegeneration via axonopathy [307]), while the toxic astrocyte phenotype, marked by pro-inflammatory factor release, directly compromises the axonal integrity [296]. Notably, combined approaches targeting the astrocyte–axon crosstalk, such as modulating astrocytic inflammation via IL-17 or propionate [299] and employing CRISPR-based correction of disease-causing mutations (e.g., in ALSP [Adult-onset Leukoencephalopathy with Axonal Spheroids and Pigmented Glia]-related genes) to concurrently ameliorate axonal pathology and astrogliosis, may be promising [308].

#### Microglial activation in axon loss and synaptopathy

Microglial activation, triggered by stressors like Aβ plaques or tau aggregates, drives disease progression in AD, PD, and other conditions by promoting phagocytosis of neurons, axonal loss, and synaptopathy [309]. Microglia directly induce axonal degeneration through inflammatory mechanisms (e.g., ET-1/ERK1/2-mediated axon retraction in optic nerve injury) and dysregulated phagocytosis of compromised axons, as evidenced in demyelinating diseases and glaucoma models, where microglial inhibition rescues axonal integrity and retinal ganglion cell survival [310–313]. Concurrently, microglia drive synaptopathy via complement (C1q/C3)-dependent and receptor-mediated phagocytosis of viable synapses, along with cytokine-induced synaptic dysfunction. This manifests as synaptic loss in the inner plexiform layer in AD models featuring "dark microglia", cognitive deficits in traumatic/sepsis models, and dendritic spine reduction in FTD. Overexpression of Transmembrane Protein 9 exacerbates the complement-mediated synaptopathy [314–318]. TREM2 dysfunction critically amplifies these pathologies: loss-of-function variants (e.g., R47H) or deletion of TREM2 decreases phosphatidylserine (PS)-dependent phagocytosis of synapses and neurons via impaired PS recognition control [319], and simultaneously disrupts binding between TREM2 and C1q and thereby unleashing complement-mediated synaptic pruning [320]. In AD models, TREM2 deficiency impairs Aβ phagocytosis, reduces plaque-associated microglial clustering, elevates tau phosphorylation, and exacerbates synaptic injury [321, 322]. Similarly, TREM2 deletion in PD accelerates α-syn propagation and dopaminergic loss [323, 324]. Mechanistically, the TREM2–DAP12 receptor complex sustains the phagocytic capacity of microglia, inhibits inflammatory hyperresponsiveness, and maintains microglial metabolic fitness and survival. However, a study in cells showed that TREM2 deficiency also enhances autophagy through the PI3K-AKT-mTOR pathway [323–325].

Therapeutically, wild-type microglia transplantation can rescue synaptic and neurodegenerative deficits [326, 327]. Therapeutic strategies targeting TREM2 function is a primary focus. Agonist antibodies activate TREM2 signaling to boost microglial phagocytosis and anti-inflammatory responses. In AD models, these antibodies (e.g., COG1410) upregulate TREM2 expression, engage the PI3K/Akt pathway, and synergistically reduce neuroinflammation while providing neuroprotection [328, 329]. However, significant challenges remain. The TREM2 antibody AL002 failed to show cognitive benefits in the INVOKE-2 trial, highlighting the uncertainty of efficacy [330]. The optimal therapeutic window remains to be defined. The context-dependent roles of TREM2—particularly its role in tau pathology (e.g., R47H loss-of-function exacerbating neurodegeneration), require deeper mechanistic understanding before targeted interventions can be optimized [322, 331, 332].

## Concluding remarks and future perspectives

This review has delineated the central role of axonopathy in neurodegenerative diseases, focusing on two critical and interconnected hubs of dysfunction: the endolysosomal trafficking system and the dynamic interplay between axons and glial cells. We have highlighted how early endosomal dysregulation, such as Rab5 hyperactivation, acts as a convergent mechanism for disrupted neurotrophic signaling, production of toxic proteins like Aβ, and impaired clearance of damaged organelles. We next explored how glial cells drive degeneration through inflammatory signaling, loss of metabolic support, and dysfunctional phagocytosis.

The SARM1-dependent NAD^+^ depletion is not an isolated phenomenon but rather a final common executioner of these upstream events. For instance, the energy crisis precipitated by mitochondrial dysfunction, whether primary or secondary to endosomal defects, directly depletes the NAD^+^ pool, lowering the threshold for SARM1 activation. Conversely, NAD^+^ supplementation strategies are inherently linked to glial function; astrocytes are key regulators of central metabolic substrates, and their ability to supply lactate or other NAD^+^ precursors could be crucial for the efficacy of such interventions. Furthermore, pro-inflammatory signals from activated microglia can exacerbate neuronal stress, potentially triggering the NAD^+^ depletion that activates SARM1. Therefore, therapeutic targeting of the SARM1/NAD^+^ axis should be viewed as complementary to strategies that rectify early endosomal dysfunction and modulate glial reactivity.

Several promising directions can be proposed for treatment of neurodegenerative diseases based on this synthesis. First, cell-type-specific therapies can be developed to switch glial activation states from a toxic to a protective phenotype, instead of broadly suppressing microglia or astrocytes. For example, ligands can be designed to selectively engage TREM2 or modulate astrocytic glutamate transporters without exacerbating inflammation. Second, the metabolic coupling between oligodendrocytes, astrocytes, and axons is a target of intervention. Enhancing lactate shuttling or other metabolic support to axons may improve their resilience to age-related stresses and transport deficits. Third, the non-cell-autonomous mechanisms require further exploration using advanced models. The integration of human iPSC-derived neurons and glia into complex 3D cultures or assembloids will enable the detailed dissection of molecular mechanisms underlying cell–cell communication in both healthy and diseased states. Finally, techniques such as in vivo imaging and spatial transcriptomics will be crucial to understanding how pathologies initiate at specific synaptic or axonal compartments and propagate through neural circuits via neuron–glia networks. In conclusion, axonal protection requires a multi-faceted approach targeting intracellular trafficking pathways and its extracellular glial environment.

## Data Availability

Not applicable.

## Abbreviations

AD: Alzheimer’s disease
PD: Parkinson’s disease
HD: Huntington’s disease
CNS: Central nervous system
AIS: Axonal initiation segment
NGF: Nerve growth factor
BDNF: Brain-derived neurotrophic factor
GDNF: Glial cell line-derived neurotrophic factor
PINK1: PTEN-induced kinase 1
VGLUT: Vesicular amino acid transporters e.g., VGLUT1
BACE1: Beta-secretase 1
NRF2: Nuclear factor erythroid 2-related factor 2
LOAD: Late onset AD
GWAS: Genome-wide association studies
RIN3: Ras and rab interactor 3
BIN1: Bridging integrator 1
CD2AP: CD2-associated protein
BFCN: Basal forebrain cholinergic neuron
HTT: Huntingtin
PFF: Pre-formed fibrils
LAG3: Lymphocyte-activation gene 3
APLP1: Amyloid precursor-like protein 1
LRRK2: Leucine-rich repeat kinase 2
iPSC: Induced pluripotent stem cell
MCTs: Monocarboxylate transporters
LDH: Lactate dehydrogenase
EAATs: Astrocytic excitatory amino acid transporters
TBI: Traumatic brain injury
GABA: Gamma-aminobutyric acid
GAT: GABA transporter
GSH: Glutathione
ROS: Reactive oxygen species
SOD: Superoxide dismutase
PRX6: Peroxiredoxin-6
IGF-1: Insulin-like growth factor 1
TREM2: Triggering receptor expressed on myeloid cells 2 protein
TDP-43: Transactive response DNA binding protein of 43 kDa
TNF-α: Tumor necrosis factor alpha
IL-1β: Interleukin-1 beta
NAD +: Nicotinamide adenine dinucleotide
FTD: Frontotemporal dementia
PS: Phosphatidylserine
mTOR: Mechanistic target of rapamycin
SARM1: Sterile alpha toll/interleukin receptor motif containing-1
